# Long-Term Adaptation of Acidophilic Archaeal Ammonia Oxidisers Following Different Soil Fertilisation Histories

**DOI:** 10.1007/s00248-021-01763-2

**Published:** 2021-05-10

**Authors:** Jun Zhao, Baozhan Wang, Xue Zhou, Mohammad Saiful Alam, Jianbo Fan, Zhiying Guo, Huimin Zhang, Cécile Gubry-Rangin, Jia Zhongjun

**Affiliations:** 1 State Key Laboratory of Soil and Sustainable Agriculture, Institute of Soil Science, Chinese Academy of Sciences, Nanjing, 210008 China; 2 School of Biological Sciences, University of Aberdeen, Cruickshank Building, St. Machar Drive, Aberdeen, AB24 3UU UK; 3 Key Lab of Microbiology for Agricultural Environment, Ministry of Agriculture, College of Life Sciences, Nanjing Agricultural University, Nanjing, 210095 China; 4 College of Agricultural Science and Engineering, Hohai University, Nanjing, 210098 China; 5 Department of Soil Science, Bangabandhu Sheikh Mujibur Rahman Agricultural University, Gazipur, 1706 Bangladesh; 6 Soil Subcenter of Chinese Ecological Research Network, Institute of Soil Science, Chinese Academy of Sciences, Nanjing, 210008 China

**Keywords:** Soil nitrification, *Nitrososphaerales*, *Ca.* Nitrosotaleaceae, *AmoA*, Low pH, Fertilisation

## Abstract

Ammonia oxidising archaea (AOA) are ecologically important nitrifiers in acidic agricultural soils. Two AOA phylogenetic clades, belonging to order-level lineages of *Nitrososphaerales* (clade C11; also classified as NS-Gamma-2.3.2) and family-level lineage of *Candidatus* Nitrosotaleaceae (clade C14; NT-Alpha-1.1.1), usually dominate AOA population in low pH soils. This study aimed to investigate the effect of different fertilisation histories on community composition and activity of acidophilic AOA in soils. High-throughput sequencing of ammonia monooxygenase gene (*amoA*) was performed on six low pH agricultural plots originating from the same soil but amended with different types of fertilisers for over 20 years and nitrification rates in those soils were measured. In these fertilised acidic soils, nitrification was likely dominated by *Nitrososphaerales* AOA and ammonia-oxidising bacteria, while *Ca.* Nitrosotaleaceae AOA activity was non-significant. Within *Nitrososphaerales* AOA, community composition differed based on the fertilisation history, with *Nitrososphaerales* C11 only representing a low proportion of the community. This study revealed that long-term soil fertilisation selects for different acidophilic nitrifier communities, potentially through soil pH change or through direct effect of nitrogen, potassium and phosphorus. Comparative community composition among the differently fertilised soils also highlighted the existence of AOA phylotypes with different levels of stability to environmental changes, contributing to the understanding of high AOA diversity maintenance in terrestrial ecosystems.

**Supplementary Information:**

The online version contains supplementary material available at 10.1007/s00248-021-01763-2.

## Introduction

**Fig. 1:**
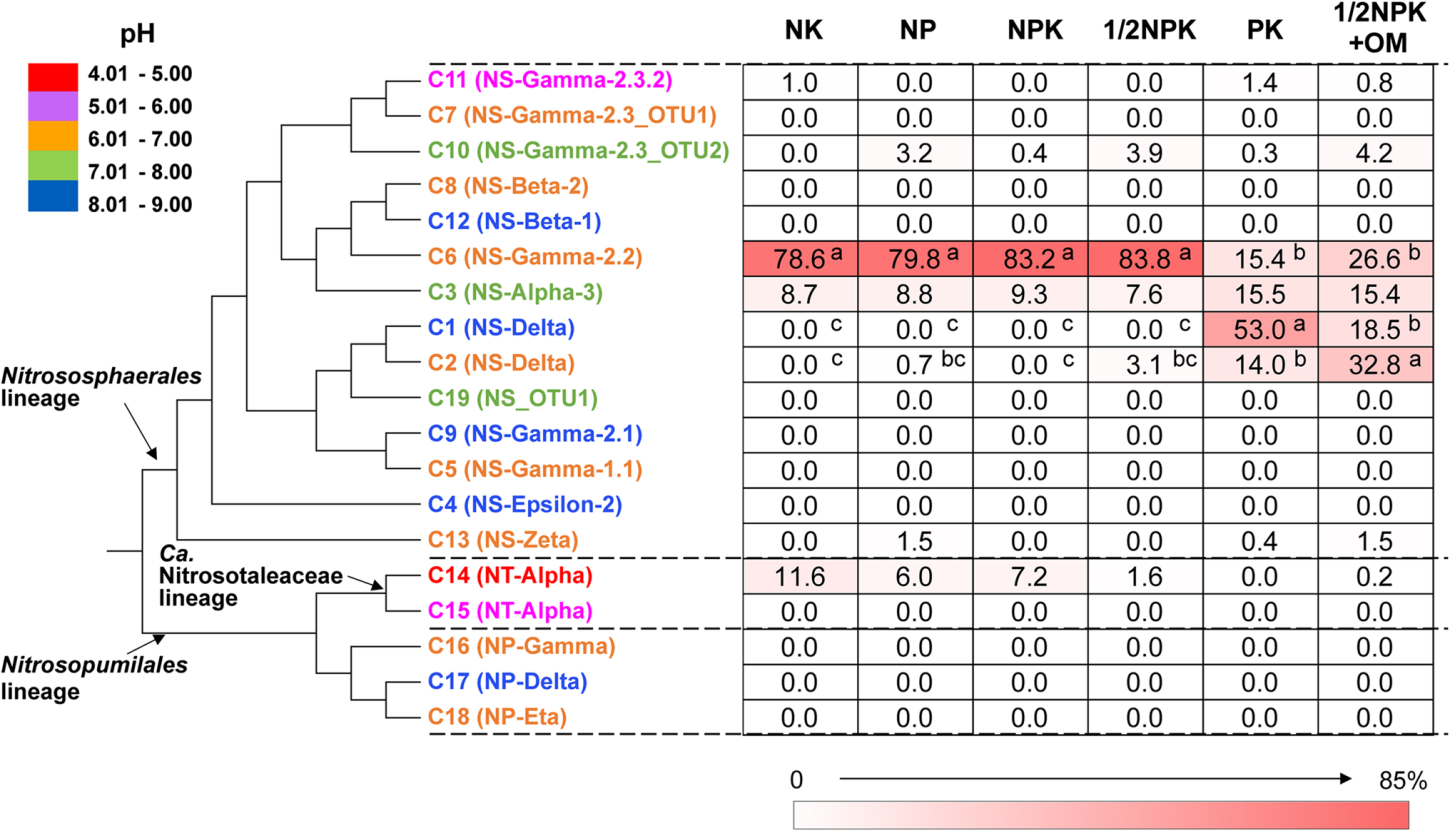
Heatmap displaying the relative abundance of different phylogenetic clades of ammonia oxidising archaea within the class *Nitrososphaeria*, estimated by pyrosequencing of archaeal *amoA* gene fragments. The phylogeny of different clades was constructed using known representative *amoA* gene sequences from each clade. Names of the phylogenetic clades include initial terrestrial denomination (C1–C19) [18] and more recent denomination of these clades is indicated in brackets [17] to unify the two phylogenetic approaches. The colour of a phylogenetic clade name indicates the pH specialisation of the clade from previous meta-analysis at a global scale [18]. Soils were ranked from low to high pH conditions (from left to right). Different letters (a–c) next to the relative abundance values for clades C1, C2 and C6 indicate significant difference in relative abundance between soils with different fertilisation history (*p* ≤ 0.05)

Acidic soils consist of almost 30% of the world’s land area and half of arable fields [1]. In the past decade, soil acidity has been intensified due to increasing amount of ammonium-based fertilisers applied in agricultural lands in China and globally [2]. Although reduced soil pH impairs many belowground biochemical processes [3], nitrification appears not to be affected as similar rates occurred in acidic and neutral soils [4]. Nevertheless, there is evidence for pH niche specialisation of ammonia oxidisers [5, 6]. The initial and rate limiting step of nitrification, the conversion of ammonia to nitrite, is performed by ammonia oxidising archaea (AOA), canonical bacteria (AOB) and more recently discovered complete ammonia bacterial oxidisers (commamox). While knowledge on comammox distribution and activity in soils is still scarce, AOA rather than AOB activity was shown to control ammonia oxidation in most of the low pH soils [7–10]. In contrast, both AOA and AOB appeared active in neutral to alkaline soils [11, 12], despite some recent evidence of AOB activity in acidic soils [13, 14]. It was further demonstrated that soil pH might determine the niche specialisation of diverse phylogenetic clades, both for AOA [5] and AOB [15]. AOA are phylogenetically placed into clades from three orders, including *Nitrososphaerales, Nitrosopumilales* and *Candidatus* Nitrosocaldales [16]. Several AOA taxonomic rankings based on the key functional gene, ammonia monooxygenase *amoA*, have been proposed in the literature and the one focusing on terrestrial AOA [5] was chosen in the present study (with correspondence with a global AOA classification [17] being presented in Fig. 1). Among the phylogenetically well characterised 19 AOA clades (cluster 1–19, C1-C19), two clades are particularly abundant in acidic and acido-neutral soils (C11 within the order-level lineage of *Nitrososphaerales* and C14 within the family-level lineage of *Ca.* Nitrosotaleaceae), while others, less abundant, also appear well-adapted to such environments (e.g. *Nitrososphaerales* C6 or some members of *Nitrososphaerales* C13) [18]. Many ecological studies have confirmed high abundance or activity of *Nitrososphaerales* and/or *Ca.* Nitrosotaleaceae AOA in acidic soils [9, 19, 20], but the environmental factors regulating their distribution are still not elucidated.

Ammonium-based N fertilisers might stimulate different groups of ammonia oxidisers, due to potential distinct affinities to and/or competitivity for substrate [21–23]. Therefore, this study aimed to test the hypothesis that different fertilisation sources also influence the AOA nitrifier community distribution in low pH agricultural soils. Indeed, it was previously observed that long-term N fertilisation affected AOA rather than AOB community compositions in low pH fields [24]. Specifically, while *Ca.* Nitrosotaleaceae AOA were observed with no amendment of nitrogen (N), phosphorus (P) or potassium (K) elements, soils amended with all those three elements (NPK) contained exclusively *Nitrososphaerales-*affiliated AOA [24]. This suggests that nutrient element limitation might be an important niche differential factor for acid-tolerant or acidophilic AOA groups, with the implication that *Nitrososphaerales* AOA are more competitive than *Ca.* Nitrosotaleaceae AOA under multiple nutrient enriched condition (i.e. fertilised with NPK or organic manure compost). To test this hypothesis, we characterised the AOA community in several low pH agricultural soils being subject to different fertilisation managements for > 20 years using high-throughput sequencing of the archaeal ammonia monooxygenase (*amoA*) gene. We predict that (i) AOA community is dominated by *Ca.* Nitrosotaleaceae and *Nitrososphaerales* AOA and more specifically by acidophilic clades of C14 and C11, and (ii) the relative abundance of Nitrososphaerales AOA will increase with multiple nutrient supplies (with NPK or manure amendment). In addition, this study aimed to identify the environmental variables potentially affecting the community composition and provide evidence for the distinct relative contribution of different ammonia oxidiser groups (*Nitrososphaerales*, *Ca. Nitrosotaleaceae* AOA and AOB) to nitrification in acidic agricultural soils under different fertilisation regimes.

## Materials and Methods

### Site Description and Soil Sampling

The long-term fertilisation experiment fields are located at the Ecology Experimental Station of Red Soil, Chinese Academy of Sciences (28° 15′ N, 116° 55′ E) in the city of Yingtan, province Jiangxi of China. This region has a typical subtropical monsoon climate with a mean annual precipitation of 1785 mm and a mean annual temperature of 18 °C. Soil derives from quaternary red clay and is classified as Hapludults. The fertilisation experiment was initially established in 1989 with a peanut-rutabaga rotation system, and since 1995 was maintained as cropping of peanut with fallow in winter. Soil samples were collected from different fields receiving different fertilisation regimes with descriptions as follows: (1) PK, plots without N fertilisation but amended with P and K; (2) NP, plots receiving chemical N fertiliser and amended with P; (3) NK, plots receiving chemical N fertiliser and amended with K; (4) NPK, plots receiving chemical N fertilisers and amended with P and K; (5) 1/2NPK, plots receiving same types of fertilisers as in NPK plots but with half amount; and (6) 1/2NPK + OM, plots receiving the same fertilisers as in 1/2NPK plots plus additional organic manure. The annual application rates of N, P and K were 120 kg urea-N/hm^2^, 40 kg P_2_O_5_-P/hm^2^ and 118 kg K_2_O-K/hm^2^, respectively, and the 1/2NPK + OM received additional 15,000 kg/hm^2^ swine manure in addition to 1/2NPK fertilisation. Each treatment was applied to three field plots and soil was collected in each plot as one biological replicate. For each biological replicate, soil samples at 0–20 cm depth were collected during fallow period within each plot from five random cores (with distance of 2–3 m between cores) in May 2012, sieved through a 2.0-mm sieve and homogenized to make a composite sample. The subsamples were kept at 4 °C until analysis for soil physiochemical analysis, and at − 20 °C for genomic DNA extraction.

### Soil Physiochemical Analysis

Soil pH was determined using a soil-to-water ratio of 1:5 with a Mettler Toledo 320-S pH meter (Mettler–Toledo Instruments Co. Ltd., Shanghai, China). Soil organic matter and total N were determined by dichromate oxidation method and Kjeldahl digestion method, respectively. Soil NH_4_^+^ and NO_3_^−^ concentrations were determined using a Skalar SAN Plus Segmented Flow Analyser (Skalar Inc., Breda, Netherlands) after extraction with 2 M KCl solution. Soil available P was extracted by sodium bicarbonate and analysed using the molybdenum blue method. Soil available K was extracted by ammonium acetate and estimated by flame photometry.

### Nucleic Acid Extraction and Pyrosequencing

Soil DNA was extracted using a FastDNA spin kit for soil (MP Biomedicals, Cleveland, OH, USA), according to the manufacturer’s instruction. The DNA quantity and purity were determined by a Nanodrop ND-1000 UV–Vis Spectrophotometer (NanoDrop Technologies, Wilmington, DE, USA) and diluted to 2–10 ng μl^−1^ for following molecular tests.

High-throughput sequencing of the *amoA* gene was performed using the primers CrenamoA 23f/CrenamoA 616r [25] with the forward primer containing unique barcode sequences for demultiplexing samples. PCR reaction was performed in a 50 µl mixture containing 25 µl of Platinum PCR SuperMix (Invitrogen, Shanghai, China), 1 µM of each primer and 2 µl of DNA template. The thermal condition was as follows: 94 °C for 3 min, 35 cycles of 94 °C for 45 s, 55 °C for 30 s and 72 °C for 90 s, followed by extension at 72 °C for 5 min. Negative control using sterile water instead of soil DNA was always included. PCR products were gel-purified and quantified using Picogreen dsDNA Quantitation Kits (Invitrogen). Adapter sequence was added to the forward end of the PCR amplicon fragments before performing the sequencing on a Roche FLX 454 pyrosequencing machine (Roche Diagnostics Corporation, Branford, CT).

The bioinformatic analyses of amplicon sequencing of marker genes included following key steps. Forward sequence reads were truncated at 350 bp and reads were demultiplexed and quality filtered using Mothur [26]. Reads with average quality score < 25, mismatched primers, ambiguous bases and frameshift errors were removed as described in a previously study [15]. The resultant sequences were assigned to a database of phylogenetic *amoA* gene clades (C1–C19) [5] using a BLASTn approach (www.ncbi.nlm.nih.gov).

### Quantification of Ammonia Oxidisers

Real-time quantitative PCR (qPCR) was performed to determine archaeal and bacterial *amoA* gene abundances on a CFX96 Optical Real-Time Detection System (Bio-Rad, Laboratories Inc., CA, USA). Primer pair Arch-amoAF/Arch-amoAR [27] was used and qPCR reaction was performed in a 25 µl volume containing 12.5 µl SYBR Premix Ex Taq (TaKaRa Biotechnology, Shiga, Japan), 1 μM of each primer and 2 μl of tenfold diluted DNA template (1–10 ng). Amplification conditions were as follows: 95 °C for 1 min, 40 cycles of 10 s at 95 °C, 30 s at 55 °C, 1 min at 72 °C, followed by plate reads at 83 °C. Bacterial *amoA* gene was quantified using the primers amoA1F/amoA2R [28], with the same PCR conditions used for archaeal *amoA* gene quantification. The standards were generated and used as previously described [8]. qPCR was performed in biological triplicate each containing 3 technical replicates. Amplification efficiency was 98–103% with *R*^*2*^ values > 0.99. The specificity of real-time PCR amplification was confirmed by melting curve analysis and agarose gel electrophoresis at the end of each qPCR run.

### Nitrification Activity by ^15^ N-Isotope Tracing Microcosms

^15^ N-isotope tracing was exploited to estimate nitrification activity under near-in situ incubation conditions. The incubation was established in triplicate in 250 ml Erlenmeyer flask containing 10 g of the sieved fresh soil from each plot. The ^15^ N labelled urea (^15^ N atom > 98%) was applied to the soil at a final concentration of 5 μg N g^−1^ dry soil, which approximated to the NH_4_^+^-N concentration in soils under in situ condition. The soil was incubated at 25 °C in darkness for 7 days in the absence or presence of 0.1% (v/v) acetylene, an inhibition gas for ammonia oxidation. The production of soil ^15^ N-labelled nitrate and nitrite (NO_x_^−^) after 7-day incubation was measured and calculated as previously described [8, 29].

### Statistical Analysis

The putative relative ammonia oxidiser contribution to ^15^NO_x_^−^ production was estimated according to the ammonia oxidiser abundance and their recorded cell-specific activity. Firstly, the relative abundance of *Nitrososphaerales* and *Ca.* Nitrosotaleaceae AOA was calculated based on high-throughput sequencing of *amoA* genes. The cell abundance of *Nitrososphaerales* and *Ca.* Nitrosotaleaceae AOA was then calculated by multiplying the relative abundance by the total AOA abundance estimated by qPCR of *amoA* genes. The cell abundance of AOB was calculated as the bacterial *amoA* gene abundance divided by 2.5, considering that one AOB cell contains on average 2.5 copies of *amoA* genes [30]. The putative proportions of *Nitrososphaerales* AOA, *Ca.* Nitrosotaleaceae AOA and AOB in the ^15^NO_x_^−^ production were then estimated according to previous record of specific cell activity of 2.6 fmol NH_3_ cell^−1^ h^−1^ for *Nitrososphaera viennensis* [31], 0.072 fmol NH_3_ cell^−1^ h^−1^ for *Ca.* Nitrosotalea devanaterra [32], and 23 fmol NH_3_ cell^−1^ h^−1^ for *Nitrosospira multiformis* [33], respectively. The specific cell activity records used here are from pure cultures under optimal cultivation conditions, and the relative contribution to the nitrification was estimated assuming they all reached these records in the soils as previously described [13].

Statistical analysis was performed on Statistics 23.0 (SPSS, IL, USA). One-way ANOVA was employed to determine the effect of different fertilisation treatments on soil ^15^NO_x_^−^ production rate and relative abundance of each AOA clade (C1-C19), followed by a Tukey post hoc test to determine significant mean differences. Alpha diversity indices, including number of OTUs and Shannon’s index, were calculated after random sampling of 400 reads per sample following OTU clustering at 100% sequence identity. Non-metric multidimensional scaling (NMDS) based on a Bray–Curtis dissimilarity matrix was performed on AOA composition using the vegan package under *R* environment, and analysis of similarity (ANOSIM) was used to assess the variations between soil sites [34]. Function ‘envfit’ was used to test the significance of chemical properties (as vectors) and fertilisation treatments (as factors) for the NMDS ordinations, with 999 permutations using vegan [34]. Differences at *p* < 0.05 were considered statistically significant.

## Results

### Changes in Soil Properties

Soil pH was affected by different fertilisation regimes. The pH was lower in most soils receiving chemical N fertilisers (pH 4.2–5.2; NK, NP, NPK and 1/2NPK) compared to the soil with no N fertilisation history (pH 5.7; PK), to the exception of the soil amended with additional supply of organic manure which had the highest pH (pH 6.3; 1/2NPK + OM) (Table 1). The soil with manure fertilisation also had the highest contents of soil organic matter (SOM), total N, inorganic N (both NH_4_^+^ and NO_x_^−^) and available P, whereas soil treatment NK had the lowest total N, inorganic N and available P concentration (Table 1). Additionally, available K contents were the highest and the lowest in PK and NP treatments, respectively (Table 1).

**Table 1:** Physiochemical properties of soils with different fertilisation history. Different letters (a–e) in each column indicate significant difference of a property between soils. *OM*, organic matter; *TN*, total N; *AP*, available P; *AK*, available K

Treatments	pH	OM (g kg^−1^)	TN (g kg^−1^)	NH_4_^+^-N (mg kg^−1^)	NO_x_^−^-N (mg kg^−1^)	AP (mg kg^−1^)	AK (mg kg^−1^)
NK	4.2^d^	12.4^b^	0.52^c^	4.8^c^	7^c^	4^e^	155^d^
NP	4.8^c^	10.7^b^	0.67^b^	5.3^b^	10^b^	46^b^	69^e^
NPK	4.9^c^	10.7^b^	0.75^b^	5.0^b^	12^b^	36^c^	249^b^
1/2NPK	5.2^c^	10.6^b^	0.66^b^	5.3^b^	11^b^	26^d^	172^c^
PK	5.7^b^	12.2^b^	0.66^b^	5.4^b^	11^b^	26^d^	335^a^
1/2NPK + OM	6.3^a^	16.4^a^	1.03^a^	6.7^a^	20^a^	319^a^	173^c^

### Compositional Change of Ammonia Oxidising Archaea

**Fig. 2:**
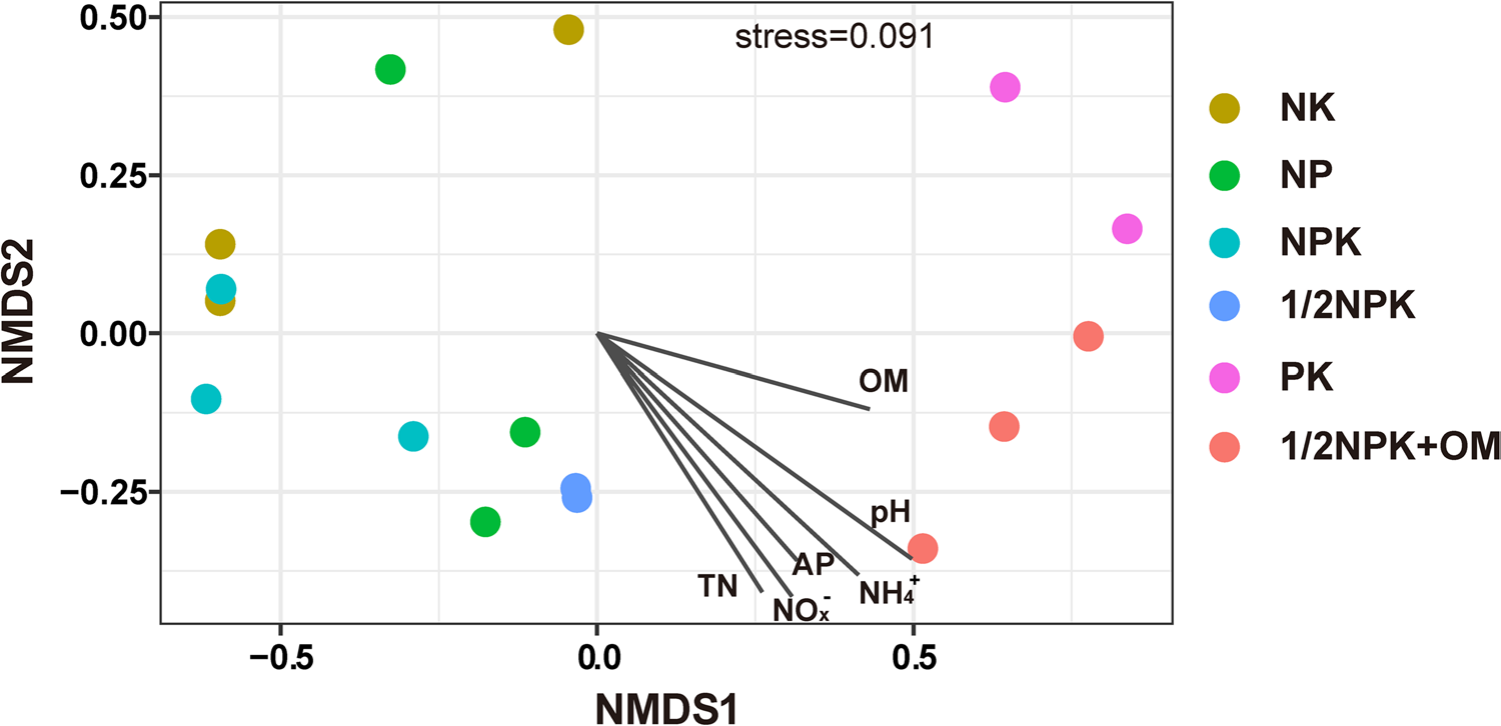
Nonmetric multidimensional scaling plot of archaeal ammonia oxidiser community compositions in different soils based on normalized abundance of different AOA clades. The soils were ranked from low to high pH conditions (from top to bottom) in the figure legend. The association of measured environmental variables was shown as the grey line segments in the plot using ‘envfit’ function, with the length of the lines proportional to the correlations between environmental variables and the ordination values and the direction pointing to increasing values of the environmental variables

Pyrosequencing of archaeal *amoA* gene fragments generated 81,958 raw sequences from all sites, with an average of 5061 raw reads (range between 2092 and 10,011 sequences) per sample. Two replicates from PK and 1/2NPK soils resulted in only 942 and 43 sequences, respectively, and were therefore removed from further analysis. Quality filtering of the sequences resulted in a total of 16,590 high-quality sequences from all sites, accounting for 20.2% of all original sequences. All AOA *amoA* sequences affiliated to the *Nitrososphaerales* and *Ca.* Nitrosotaleaceae lineages (Fig. 1 and Fig. S1), with *Nitrososphaerales* accounting for 88.4–100% of *amoA* gene sequences in different soils. *Nitrososphaerales* C6, C1, C2 and C3 clades were the most abundant phylogenetic clades accounting for up to 83.8%, 53.0%, 32.8% and 15.5% of the AOA population, respectively (Fig. 1). In contrast, *Ca.* Nitrosotaleaceae C14 accounted for up to 11.6% of the AOA population (Fig. 1). Within *Nitrososphaerales*, the relative abundance of the C1, C2 and C6 clades depended on the soil fertilisation histories, with C1 and C2 having the highest relative abundance in soils with no N fertilisation (PK) or extra organic manure fertilisation (1/2NPK + OM), which were the soils with the highest pH (Table 1), while C6 relative abundance was highest in the other soils (Fig. 1). Due to the shifts in the relative abundance of different AOA clades, AOA compositions were dissimilar in soils with different fertilisation histories, based on NMDS analysis (Fig. 2). Different fertilisation treatments had a significant effect on the AOA community compositions between higher (PK and 1/2NPK + OM, pH 5.7–6.3) and lower (NK, NP, NPK and 1/2NPK, pH 4.2–5.2) pH soil groups (*p* = 0.002) following ANOSIM analysis based on the Bray–Curtis dissimilarity. Multivariate analyses showed that change in soil pH most strongly correlated with variation in AOA compositions among all tested environmental variables (Fig. 2 and Table S1). The alpha diversity indices of AOA, including richness (number of OTUs) and evenness (Shannon’s index), were higher in PK and 1/2NPK + OM soils than the other soils, although the difference was not significant for the richness index (Fig. S2).

### Ammonia Oxidiser Abundance

**Fig. 3:**
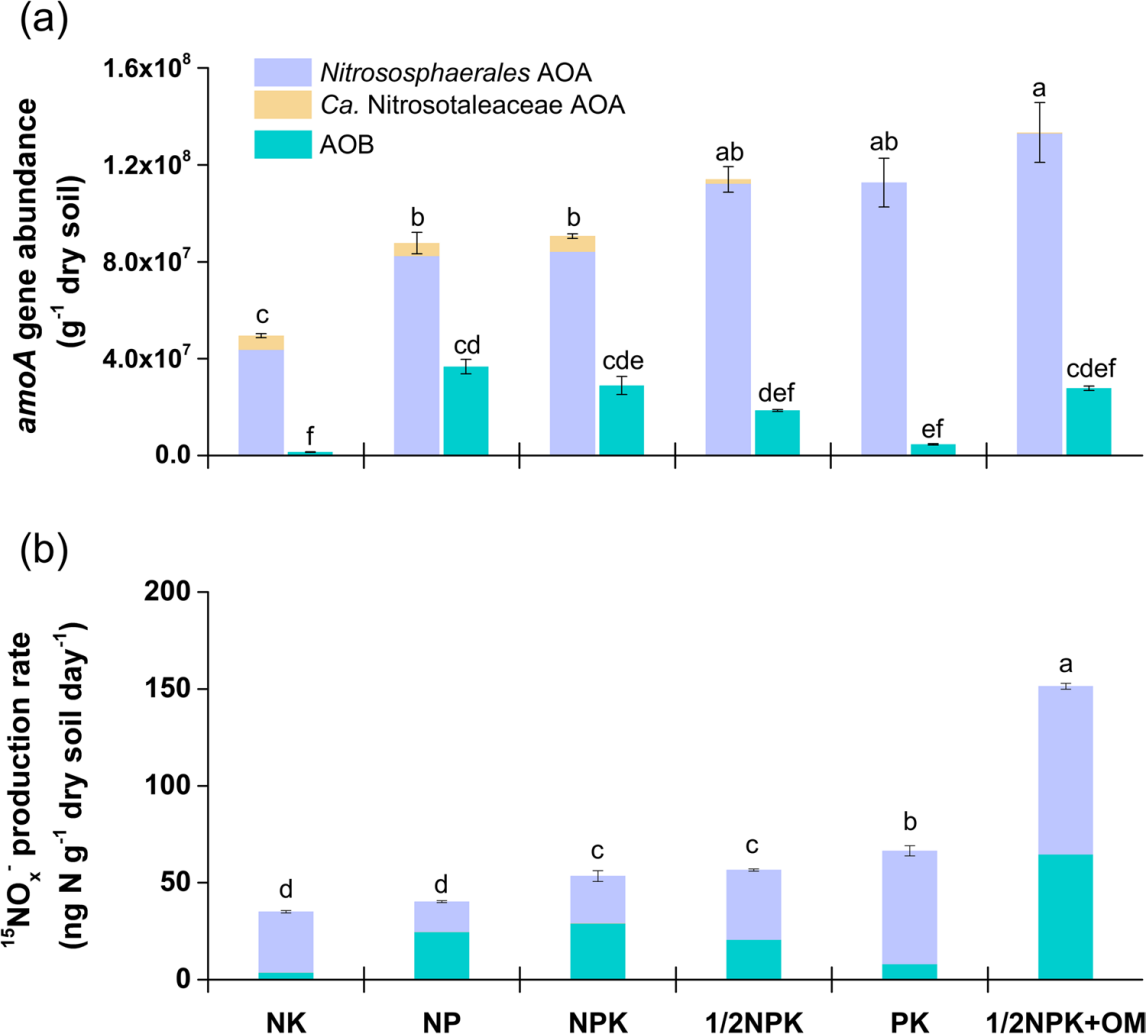
Abundance (**a**) and putative contribution (**b**) of *Nitrososphaerales* AOA, *Ca.* Nitrosotaleaceae AOA and betaproteobacterial AOB to nitrification. **a** Total AOA and AOB abundances were estimated by qPCR of *amoA* genes, and *Nitrososphaerales* AOA and *Ca.* Nitrosotaleaceae AOA abundances were calculated by multiplying total AOA abundance by the relative abundance of each AOA lineage. **b** Nitrification activity was estimated as the production rate of ^15^NO_x_^−^-N converted from ^15^ N-urea following 7-day microcosm incubation and the putative contributions were estimated using specific cell activity of 2.6, 0.072 and 23 fmol NH_3_ cell^−1^ h^−1^ for *Nitrososphaerales* AOA, *Ca.* Nitrosotaleaceae AOA and AOB, respectively. Soils were ranked from low to high pH conditions (from left to right). Error bars represent standard errors of means from triplicate microcosms and different letters above the bars indicate statistically significant differences (*p* ≤ 0.05) within each plot

Both AOA and AOB were detected in all soils by quantification of *amoA* genes. AOA gene abundance ranged between 4.9 × 10^7^ and 1.3 × 10^8^ per g of soil, with the highest abundance being estimated in the soils with higher pH (Fig. 3a). After multiplying the total AOA gene abundance by the relative abundance of *Nitrososphaerales* and *Ca.* Nitrosotaleaceae (based on the pyrosequencing analysis), the abundance of these two clades was estimated to be 4.4 × 10^7^–1.3 × 10^8^ and 0–6.6 × 10^6^ per g of soil, respectively (Fig. 3a). AOB gene abundance was significantly lower than AOA abundance in all soils, ranging from 1.4 × 10^6^ to 3.7 × 10^7^ per g of soil (Fig. 3a).

### Nitrification Activity and Putative AO Contributions to Soil Nitrification

Soil nitrification activity was assessed by temporal increase in ^15^ N-NO_x_^−^ content converted from ^15^ N-NH_4_^+^ amended in the microcosms after 7-day incubation. The highest nitrification activity was observed in the soil receiving organic manure (1/2NPK + OM treatment), while the lowest activity was detected in NK and NP soils (Fig. 3b). Acetylene fully inhibited nitrate production in all soil microcosms (Fig. S3). Putative ammonia oxidiser contributions to the nitrification rate were then estimated according to the ammonia oxidiser abundance and the recorded AOA and AOB cell-specific activity. *Nitrososphaerales* AOA and AOB were putatively the major contributors to nitrification in these soils, accounting for 38.9–89.5% and 10.1–60.9% of the nitrification rates, respectively, while *Ca. Nitrosotaleaceae* AOA contribution was negligible (0–0.4% of the rates) (Fig. 3b).

**Fig. 4:**
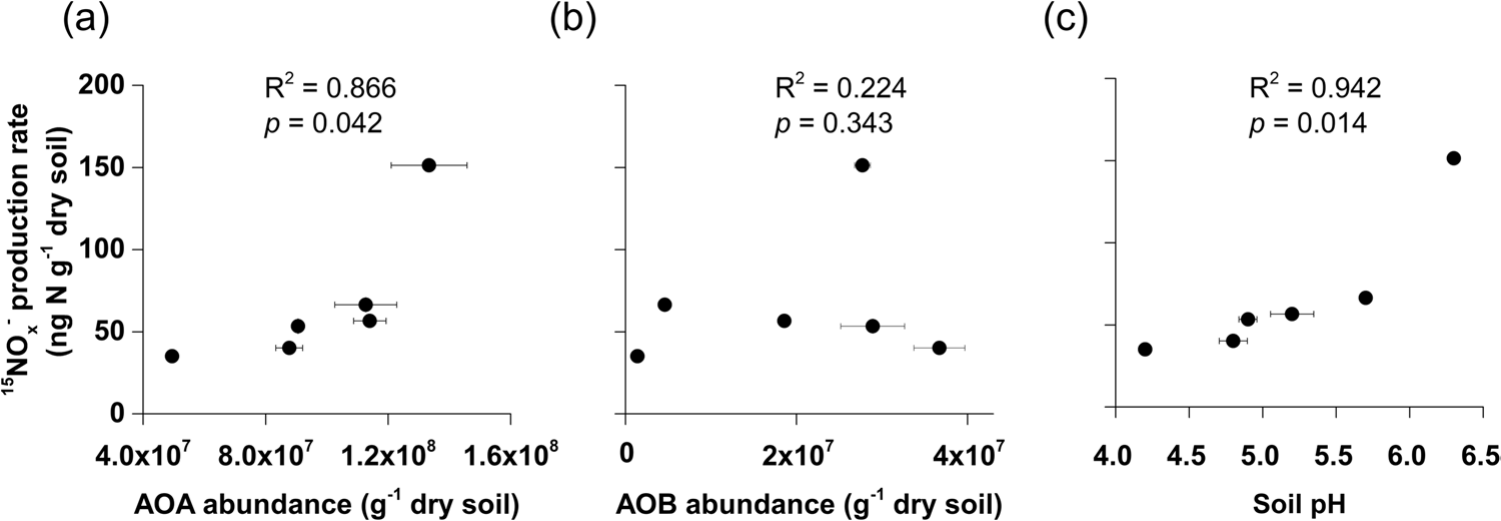
Correlations of ^15^ N-NO_3_^−^ production rate with AOA abundance (**a**), AOB abundance (**b**) and soil pH (**c**). AOA and AOB abundances were estimated by qPCR of archaeal and bacterial *amoA* genes, respectively. Error bars represent standard errors of means from triplicate microcosms and regression coefficients of the best fitting model and associated *p* values are indicated. Small standard error values were hidden by the mean value symbols

Soil ^15^NO_x_^−^ production rate was positively correlated with archaeal *amoA* gene abundance (polynomial best-fitting model: *y* = 3e^−14^*x*^2^ – 4e^−06^*x* + 173, *p* = 0.042) (Fig. 4b) but not with bacterial *amoA* gene abundance (*p* = 0.343) (Fig. 4b). Soil ^15^NO_x_^−^ production rate also positively correlated with soil pH (polynomial best-fitting model: *y* = 3*4x*^2^ – 304*x* + 723, *p* = 0.014) (Fig. 4c). Additionally, soil pH positively correlated with AOA abundance but not AOB abundance (Fig. S4).

## Discussion

### The Ecology of *Nitrososphaerales* and *Ca*. *Nitrosotaleaceae* AOA in Acidic Agricultural Soils

This study predicted that *Nitrososphaerales* and *Ca.* Nitrosotaleaceae clades dominate AOA population in the low pH agricultural soils, especially the clades of C11 and C14, which are the most abundant AOA clades in acidic soils based on a global database study [5, 35]. Unexpectedly, neither C11 nor C14 was retrieved as the main AOA nitrifiers in our soils, indicating that strong physiological adaptation to low soil pH was not restricted to these previously recognized AOA clades. Nonetheless, the relative abundance of C14 seemed to increase in the soils with a decreased pH (especially in NK soil, pH 4.2, Fig. 1), despite no statistical difference observed between different soils due to large replication variations in this study. This suggested great adaptability of C14 to strongly acidic environment (pH < 4.5), being consistent with previous studies showing strong AO activity of this clade in such soils (see list of SIP experiments in Table 2). Additionally, previous studies on acidic red soils from the same region (Yingtan, see Table 2) estimated that *Nitrososphaerales* C11 were the most abundant AOA in some forest soils while they were not detected in reclaimed agricultural soils, suggesting that this AOA clade might not be favoured under agricultural management. This might explain the generally low proportion of *Nitrososphaerales* C11 in all agricultural soils used in this study.

**Table 2:** Non-restrictive meta-analysis of archaeal *amoA* gene communities in 35 acidic soils across China from 15 different studies. Studies were selected following the key topic word search ‘amoA archaea acidic soil’ in Web of Science. After manual curation, only studies on acidic agricultural soils with *amoA* sequencing data were included in the list for comparison with the present study. The archaeal *amoA* gene sequences were retrieved from GenBank and classified into 19 phylogenetic clades [5] by BLASTn approach. The table indicates the relative abundance of the main clades for each study when such estimation was possible. The full literature list is shown in supplementary Table S2

Soil site	Source	pH	Main clade(s)	Analysis tool	Presence or active	literature
Studies on different fertilisation histories						
1. Qiyang (Hunan)	Agricultural soil (N)	3.7	C14/C15 (55%), C6 (45%)	Clone library	Presence	He et al. 2007
	Agricultural soil (NK)	3.8	C14/C15 (82%), C3/C6/C11 (18%)			
	Agricultural soil (NP)	4.0	C14/C15 (77%), C2/C6 (23%)			
	Agricultural soil (NPK)	4.0	C6 (100%)			
	Agricultural soil (PK)	5.0	C14/C15 (12.5%) C1/C3/C6/C10/C11 (87.5%)			
	Agricultural soil (no fertiliser)	5.5	C1/C3/C6/C10 (100%)			
	Agricultural soil (NPK + OM)	5.8	C1/C6/C10 (100%)			
	Agricultural soil (Fallow)	5.8	C1/C3 (100%)			
2. Nanchang (Jiangxi)	Agricultural soil (N)	5.3	C6	DGGE/Clone library	Presence	Shen et al. 2015
	Agricultural soil (CK)	5.4	C6/C10			
	Agricultural soil (NPK)	5.8	C6/C10			
	Agricultural soil (OM)	5.8	C1/C2			
	Agricultural soil (NPK + OM)	5.9	C1/C2			
Studies on Yingtan soil						
3. Yingtan (Jiangxi)	Broad-leaf forest	4.3	C11	DGGE/Clone library	Presence	Huang et al. 2011
	Bush forest	4.4	C11/C14			
	Peanut soil	4.8	C3/C6			
4. Yingtan (Jiangxi)	Broad-leaf forest	4.4	C11	Meta-genome	Presence	Wang et al. 2019
5. Yingtan (Jiangxi)	Agricultural soil	5.0	C3/C13	T-RFLP/Clone library	Presence	Wu and Conrad 2014
6. Yingtan (Jiangxi)	Agricultural soil	4.9	C3 (100%)	DNA-SIP/Clone library	Active	Wang et al. 2014a
Other studies						
7. Hangzhou (Zhejiang)	Tea orchard	3.8	C14/C15	DNA-SIP/Clone library	Active	Lu and Jia 2013
8. Hangzhou (Zhejiang)	Tea orchard	3.8	C14	DNA-SIP/Clone library	Active	Wang et al. 2019
9. Taoyuan (Hunan)	Corn/seed rape	4.0	C14	DGGE/Clone library	Presence	Shen et al. 2013
10. Hangzhou (Zhejiang)	Tea orchard	4.2	C11/C15	DNA-SIP/Clone library	Active	Zhang et al. 2012
11. Ningbo (Zhejiang)	Vegetable soil (Native pH)	4.0	C3 (97.5%)	DNA-SIP/Clone library	Active	Li et al. 2019
	Vegetable soil (modified pH)	4.8	C3 (38%), C1 (35%), C2(17%)			
12. Hefei (Anhui)	Vegetable soil	4.3–6.3	C14	454-pyrosequencing	Presence	Song et al. 2016
	Vegetable soil	7.0	C3			
13. Jiansanjiang (Heilongjiang)	Soybean field	4.5	C11	Clone library	Presence	Wang et al. 2014b
14. Ji’an (Jiangxi)	Citrus field (fertilisation)	4.7–5.1	C3/C14	TFRLP/Clone library	Presence	Liu et al. 2017
	Citrus field (no fertilisation)	5.0–5.1	C14			
15. Shenyang (Liaoning)	Agricultural soil (high N fertilisation)	5.2	C1/C14	DGGE/Clone library	Presence	Xu et al. 2012
	Agricultural soil (medium N fertilisation)	5.5	C1/C3			
	Agricultural soil (low N fertilisation)	5.6	C1/C2/C3			
	Agricultural soil (no fertiliser)	5.7	C1/C2			

Instead of *Nitrososphaerales* C11 and *Ca*. Nitrosotaleaceae C14, AOA population was dominated by *Nitrososphaerales* C3, C6 or C1/C2 clades in our soils. However, these AOA clades had different levels of adaptability to different fertilisation histories, supposedly leading to distinct AOA compositions in these soils (Fig. 2). *Nitrososphaerales* clade C3 were detected at similar proportions in different soils, irrespective of different fertilisation history (Fig. 1 and Fig. S1), suggesting nitrifiers within this clade adapt to a wide range of environmental conditions and form the common and widespread archaeal nitrifiers in these soils. This consistently detected AOA clade might be considered ‘habitat generalist’ with similar distribution pattern in these soils [36], which can play a key role in maintenance of taxonomic diversity [37] and may have strong potential for dormancy [38]. In comparison, C6 and C1/2 showed higher sensitivity to environmental changes, as C6 were most abundant in lower pH soils (NK, NP, NPK and 1/2NPK, pH 4.2–5.2) and C1/2 dominated in more neutral pH soils (PK and 1/2NPK + OM, pH 5.7–6.3) (Fig. 1 and Fig. S1). The compensation of C1/C2 abundance by C6 in lower pH soils suggests redundant function of distinct AOA phylotypes in soils, which might facilitate fast recovery of nitrification rate following environmental changes [39]. Similar trend was observed from a list of literatures (Table 2), as C1/C2 clades only dominated AOA population in soils with pH ≥ 5.7 and the C6 were often predominant in lower pH soils (4.0–5.3). In addition, *Nitrososphaerales* clade C10 was abundantly detected in many lower pH soils from previous studies, which was not observed in our soils. Interestingly, based on DNA-based stable isotope probing studies, the most actively growing *Nitrososphaerales* clades in these low pH agricultural soils were restricted to clades of C1/C2, C3 and C11 (Table 2) [9, 19, 40]. This indicated the lifestyles and metabolic traits of different AOA clades might be distinct. While millions years of evolution separate these AOA clades and associated metabolic traits [5, 35], AOA adaptation to such acidic conditions was likely facilitated by acquisition of V-type ATPases via horizontal transfer [41] or other traits linked to pH homeostasis [42].

### Influence of Different Fertilisation Histories on AOA Composition and Abundance

We also hypothesized that proportion of *Nitrososphaerales* AOA would be greater in the low pH agricultural soils receiving higher nutrient supplies of NPK or manure amendment. However, this study did not observe significant difference of *Nitrososphaerales* proportions between soils receiving all major nutrient elements (NPK, 1/2NPK and 1/2NPK + OM) and those with one nutrient element unamended (NP, NK, PK). Different nutrient amendments appeared to have no direct impact on the AOA community compositions, but the consequent change in soil pH condition might be one of the key drivers, according to correlation test on several environmental variables (Fig. 2). Indeed, pH could roughly classify the soils into two main groups, one with more acidic pH range (NK, NP, NPK and 1/2NPK, pH ≤ 5.2) having closer AOA community structure compared to the higher pH soil group (PK and 1/2NPK + OM, pH 5.7–6.3) (Fig. 2). These two groups of soils were also dominated by distinct AOA clades as discussed above and had different AOA diversity indices (Fig. S2). Some nutrient factors, e.g. soil NH_4_^+^ concentration, also showed consistency with AOA composition based on multivariate analyses (Fig. 2). However, their role in shaping AOA community assembly is uncertain, as most of these factors are fluctuating with agricultural management.

Although the multiple nutrient supplies might not determine the composition of AOA in our soils, the amendment or lack of certain nutrient element might affect the ammonia oxidiser activity. For instance, there was no significant difference observed in soil pH (Table 1) and ammonia oxidiser abundance (Fig. 3a) between NP, NPK and 1/2NPK treatments, but the nitrification rate was the lowest in NP soil without K supply history, suggesting that increased K supply could be a factor enhancing the ammonia oxidiser activity. Additionally, the only soil without P fertiliser application (NK) had both the lowest ammonia oxidiser abundance and nitrification rate. Since NK soil also had the lowest pH, we cannot disentangle the influence of P element from the pH change. However, an enhanced microbial N cycling, including nitrification process, was previously observed following P addition in agricultural soils [43, 44]. Lastly, organic manure fertilisation history also had significant effect on the AOA composition (Table S1) and the soil receiving such fertilisation had the highest nitrification rate in our study (Fig. 3). Because this soil also had the highest pH, it is difficult to distinguish which factor is more determinant of such observation. Nevertheless, a previous study observed that organic mature fertilisation induced an increased nitrification rate without a change in soil pH [45], implying that other environmental elements important for ammonia oxidiser activity might have been introduced in soils following mature fertilisation regime. Indeed, the soil fertilised with manure accumulated the highest SOM content among all soils used in this study (Table 1) and such SOM concentration might favour high AOA growth, as AOA grow well under supply of ammonia originating from organic N mineralisation [22, 46].

## Conclusion

The present study revealed a high abundance of *Nitrososphaerales* AOA in typical Chinese acidic red soils following different agricultural managements. Different long-term fertilisation regimes applied to the soils altered the relative abundance of several major *Nitrososphaerales* clades in soils, i.e. C1, C2 and C6, likely through perturbation of soil pH. Our results suggested that the high abundance and associated physiological adaptation to these low soil pH conditions were not restricted to previously recognized AOA clades, i.e. *Ca. Nitrosotaleaceae* C14 and *Nitrososphaerales* C11. The major *Nitrososphaerales* clades identified in our soils are potentially responsible for the in situ ammonia oxidation process, but assessment of their activity and relative contribution requires further investigation using high sensitivity techniques, such as DNA-stable isotope probing or RNA-based tools.

## Supplementary Information

Below is the link to the electronic supplementary material.

Supplementary file1 (DOCX 322 KB)

## Data Availability

The pyrosequencing reads of archaeal *amoA* genes have been deposited at European Nucleotide Archive (ENA) with accession number PRJEB40021.
