# Supplementary material for: Long-Term Adaptation of Acidophilic Archaeal Ammonia Oxidisers Following Different Soil Fertilisation Histories
Source: Microb Ecol. 2021 May 10;83(2):424–35. doi: 10.1007/s00248-021-01763-2 (PMC8891100; doi:10.1007/s00248-021-01763-2)
Supplement: Supplementary file 1 — Supplementary file1 (DOCX 322 KB) [file 248_2021_1763_MOESM1_ESM.docx]

**Supplementary material for:**

**Long-term adaptation of** **acidophilic archaeal ammonia oxidisers following different soil fertilisation histories**

Jun Zhao^1,2†^, Baozhan Wang^1,3†^, Xue Zhou^4^, Mohammad Saiful Alam^1,5^, Jianbo Fan^1^, Zhiying Guo^6^, Huimin Zhang^1^, Cécile Gubry-Rangin^2*^ and Zhongjun Jia^1*^

^1^ State Key Laboratory of Soil and Sustainable Agriculture, Institute of Soil Science, Chinese Academy of Sciences, Nanjing, 210008, China

^2^School of Biological Sciences, University of Aberdeen, Cruickshank Building, St. Machar Drive, Aberdeen, AB24 3UU, UK

^3^Key Lab of Microbiology for Agricultural Environment, Ministry of Agriculture, College of Life Sciences, Nanjing Agricultural University, Nanjing, 210095, China

^4^College of agricultural science and engineering, Hohai University, Nanjing, 210098, China

^5^Department of Soil Science, Bangabandhu Sheikh Mujibur Rahman Agricultural University, Gazipur-1706, Bangladesh

^6^Soil Subcenter of Chinese Ecological Research Network, Institute of Soil Science, Chinese Academy of Sciences, Nanjing, 210008, China

^*^Corresponding authors: Cécile Gubry-Rangin (c.rangin@abdn.ac.uk); Zhongjun Jia (jia@issas.ac.cn)

^†^These authors contributed equally to this work





**Figure S1.** Relative abundances of *Nitrososphaerales* and *Candidatus* Nitrosotaleales AOA in different soils. *Ca.* Nitrosotaleales AOA were exclusively represented by C14 clade. The soils were ranked from low to high pH conditions (from top to bottom). Error bars represent standard errors of means from triplicate microcosms and different letters next to the bars indicate statistically significant differences (*p*≤0.05) within each plot.





**Figure S2.** Richness (a) and evenness (b) of ammonia oxidizing archaea in soils. The indices were calculated with random sampling with depth of 400 reads per soil and OTUs were clustered at 100% identity after removal of singleton sequences. Error bars represent standard errors of means of replication and different letters above the bars in each plot indicate statistically significant differences (*p*≤0.05).





**Figure S3.** The ^15^NO_x_^-^ content in the soils before (Day 0) and after 7-day incubation of soil microcosms amended with ^15^N-urea in the absence (Day 7) or presence of nitrification inhibitor acetylene (Day 7 + C_2_H_2_). Error bars represent standard errors of means from triplicate microcosms and different letters above the bars in each soil indicate statistically significant differences (*p*≤0.05).





**Figure S4.** Correlations of soil pH with AOA and AOB abundances. AOA and AOB abundances were estimated by qPCR of archaeal and bacterial *amoA* genes, respectively. Error bars represent standard errors of means from triplicate microcosms and regression coefficients of the best fitting model and associated *p* values are indicated.

**Table S1** Correlation of environmental variables with AOA community compositions following envfit procedure. Pr(>r) indicates the level of significance based on 999 random permutations. Abbreviations: TN, total N; AP, Available P; AK, Available K. OM content, organic matter content; OM fertiliser, organic manure fertiliser.

| Variables | r2 | Pr(>r) |
| --- | --- | --- |
| Vectors (chemical properties) | |  |
| pH value | 0.8131 | 0.001 |
| OM content | 0.4342 | 0.021 |
| TN content | 0.5109 | 0.010 |
| NH_4_^+^ content | 0.6870 | 0.001 |
| NO_x_^-^ content | 0.5813 | 0.004 |
| AP content | 0.4968 | 0.013 |
| AK content | 0.1526 | 0.351 |
| Factors (fertilisation treatments) | | |
| K fertiliser | 0.0305 | 0.613 |
| P fertiliser | 0.1590 | 0.103 |
| NK fertiliser | 0.0582 | 0.378 |
| NP fertiliser | 0.0891 | 0.261 |
| PK fertiliser | 0.2001 | 0.067 |
| NPK fertiliser | 0.0760 | 0.285 |
| OM fertiliser | 0.3201 | 0.017 |

**Table S2** List of literatures used in Table 2.

| Study No. | Reference |
| --- | --- |
| 1 | He JZ, Shen JP, Zhang LM, Zhu YG, Zheng YM, Xu MG, Di H (2007) Quantitative analyses of the abundance and composition of ammonia-oxidizing bacteria and ammonia-oxidizing archaea of a Chinese upland red soil under long-term fertilization practices. Environ Microbiol 9:2364-2374. <https://doi.org/10.1111/j.1462-2920.2007.01358>.x |
| 2 | Huang R, Wu Y, Zhang J, Zhong W, Jia Z, Cai Z (2011) Nitrification activity and putative ammonia-oxidizing archaea in acidic red soils. J Soil Sediment 12:420-428. https://doi.org/10.1007/s11368-011-0450-4 |
| 3 | Li Y, Xi R, Wang W, Yao H (2019) The relative contribution of nitrifiers to autotrophic nitrification across a pH-gradient in a vegetable cropped soil. J Soil Sediment 19:1416-1426. https://doi.org/10.1007/s11368-018-2109-x |
| 4 | Liu H, Wu X, Wang Q, Wang S, Liu D, Liu G (2017) Responses of soil ammonia oxidation and ammonia-oxidizing communities to land-use conversion and fertilization in an acidic red soil of southern China. Eur J Soil Biol 80:110-120. https://doi.org/10.1016/j.ejsobi.2017.05.005 |
| 5 | Lu L, Jia Z (2013) Urease gene-containing Archaea dominate autotrophic ammonia oxidation in two acid soils Environ Microbiol 15:1795-1809. https://doi.org/10.1111/1462-2920.12071 |
| 6 | Shen JP, Cao P, Hu HW, He JZ (2013) Differential response of archaeal groups to land use change in an acidic red soil. Sci Total Environ 461-462:742-749. https://doi.org/10.1016/j.scitotenv.2013.05.070 |
| 7 | Shen W, Xu T, Liu J, Huang Q, Gu G, Zhong W (2015) Long-term application of organic manure changes abundance and composition of ammonia-oxidizing archaea in an acidic red soil. Soil Sci Plant Nutr 61:620-628. https://doi.org/10.1080/00380768.2015.1023687 |
| 8 | Song H, Che Z, Cao W, Huang T, Wang J, Dong Z (2016) Changing roles of ammonia-oxidizing bacteria and archaea in a continuously acidifying soil caused by over-fertilization with nitrogen. Environ Sci Pollut Res Int 23:11964-11974. https://doi.org/10.1007/s11356-016-6396-8 |
| 9 | Wang B et al. (2019) Expansion of Thaumarchaeota habitat range is correlated with horizontal transfer of ATPase operons. ISME J 13:3067-3079. https://doi.org/10.1038/s41396-019-0493-x |
| 10 | Wang B, Zheng Y, Huang R, Zhou X, Wang D, He Y, Jia Z (2014a) Active ammonia oxidizers in an acidic soil are phylogenetically closely related to neutrophilic archaeon. Appl Environ Microb 80:1684-1691. https://doi.org/10.1128/AEM.03633-13 |
| 11 | Wang J, Wang W, Gu JD (2014b) Community structure and abundance of ammonia-oxidizing archaea and bacteria after conversion from soybean to rice paddy in albic soils of Northeast China. Appl Microbiol Biotechnol 98:2765-2778. https://doi.org/10.1007/s00253-013-5213-2 |
| 12 | Wu Y, Conrad R (2014) Ammonia oxidation-dependent growth of group I.1b Thaumarchaeota in acidic red soil microcosms. FEMS Microbiol Ecol 89:127-134. https://doi.org/10.1111/1574-6941.12340 |
| 13 | Xu Y-G, Yu W-T, Ma Q, Zhou H (2012) Responses of bacterial and archaeal ammonia oxidisers of an acidic luvisols soil to different nitrogen fertilization rates after 9 years. Biol Fertil Soils 48:827-837. https://doi.org/10.1007/s00374-012-0677-2 |
| 14 | Zhang L-M, Hu H-W, Shen J-P, He J-Z (2012) Ammonia-oxidizing archaea have more important role than ammonia-oxidizing bacteria in ammonia oxidation of strongly acidic soils. ISME J 6:1032-1045. https://doi.org/10.1038/ismej.2011.168 |
